# The Red Herring Cast: Interstitial Nephritis in Disguise

**DOI:** 10.7759/cureus.96894

**Published:** 2025-11-15

**Authors:** Anagha Auradkar

**Affiliations:** 1 Nephrology, Dr. B.R. Ambedkar Medical College, Bangalore, IND

**Keywords:** acute kidney injury, diabetic nephropathy, interstitial nephritis, kidney biopsy, nephritic syndrome, rapidly progressive glomerulonephritis, tubulointerstitial disease

## Abstract

A 36-year-old man with a 12-year history of type 2 diabetes mellitus and recently diagnosed hypertension presented with oliguria, edema, and rapidly progressive renal dysfunction. Urinalysis revealed nephritic sediment with significant proteinuria, red blood cells (RBCs), and cellular casts, raising a strong suspicion for rapidly progressive glomerulonephritis. However, renal biopsy showed acute on chronic interstitial nephritis characterized by dense lymphoplasmacytic infiltrates, tubular atrophy, regenerative atypia, and moderate interstitial fibrosis, without crescents or immune deposits. This case highlights the diagnostic challenge of acute interstitial nephritis (AIN) mimicking glomerular disease in diabetic patients. Early biopsy was essential to avoid misclassification and inappropriate immunosuppression. The report underscores the importance of considering interstitial pathology in atypical or rapidly worsening renal failure among diabetics and reinforces the role of kidney biopsy as the gold standard for accurate diagnosis and appropriate management.

## Introduction

Acute interstitial nephritis (AIN) is a potentially reversible cause of acute kidney injury that can present with diverse and sometimes misleading clinical features. In certain cases, AIN can closely mimic rapidly progressive glomerulonephritis (RPGN), especially when patients exhibit active urinary sediment with RBCs, proteinuria, and casts. This overlap creates a significant diagnostic challenge, as the treatment strategies for these two conditions are very different. This report describes a case of AIN in a diabetic patient that initially resembled RPGN, highlighting the critical role of renal biopsy in establishing an accurate diagnosis and guiding appropriate management.

## Case presentation

A 36-year-old man with a 12-year history of type 2 diabetes mellitus and recently diagnosed hypertension presented with reduced urine output and swelling of the legs. On examination, he had mild pedal edema and stable vital signs.

Laboratory investigations revealed a serum creatinine of 2.44 mg/dL at admission, which rose to 5.2 mg/dL over the following days. Complete blood count showed leukocytosis with neutrophilia. Urinalysis demonstrated 3+ proteinuria, 11-12 RBCs per high-power field, and the presence of both red blood cell (RBC) and white blood cell (WBC) casts. Serological evaluation was performed in this patient: antinuclear antibody (ANA) was negative, and complement levels (C3, C4) were within normal limits. Antineutrophil cytoplasmic antibody (ANCA) testing was not performed as the patient was unable to afford it. These findings, together with oliguria and rapid deterioration of renal function, initially raised strong concern for RPGN. Laboratory results are summarized in Table 1.

**Table 1:** Laboratory investigations at presentation Laboratory investigations showed rising creatinine and uremia with leukocytosis and neutrophilia. Urinalysis demonstrated heavy proteinuria, hematuria with dysmorphic RBCs, and both RBC and WBC casts, suggestive of nephritic sediment. BUN: blood urea nitrogen; WBC: white blood cell; RBC: red blood cell

Parameter	Result	Reference range
Serum creatinine	2.44→5.2 mg/dL	0.6-1.2 mg/dL
BUN	68 mg/dL	7-20 mg/dL
Hemoglobin	11.2 g/dL	12-6 g/dL
WBC count	8130/mm³	4000-11000/mm³
Neutrophils	75%	40%-70%
Platelet count	154000/mm³	150000-450000/mm³
Urine protein	3+	Negative
Urine RBCs	11-12/hpf	0-2/hpf
RBC casts	Present	Absent
WBC casts	Present	Absent

**Figure 1:**
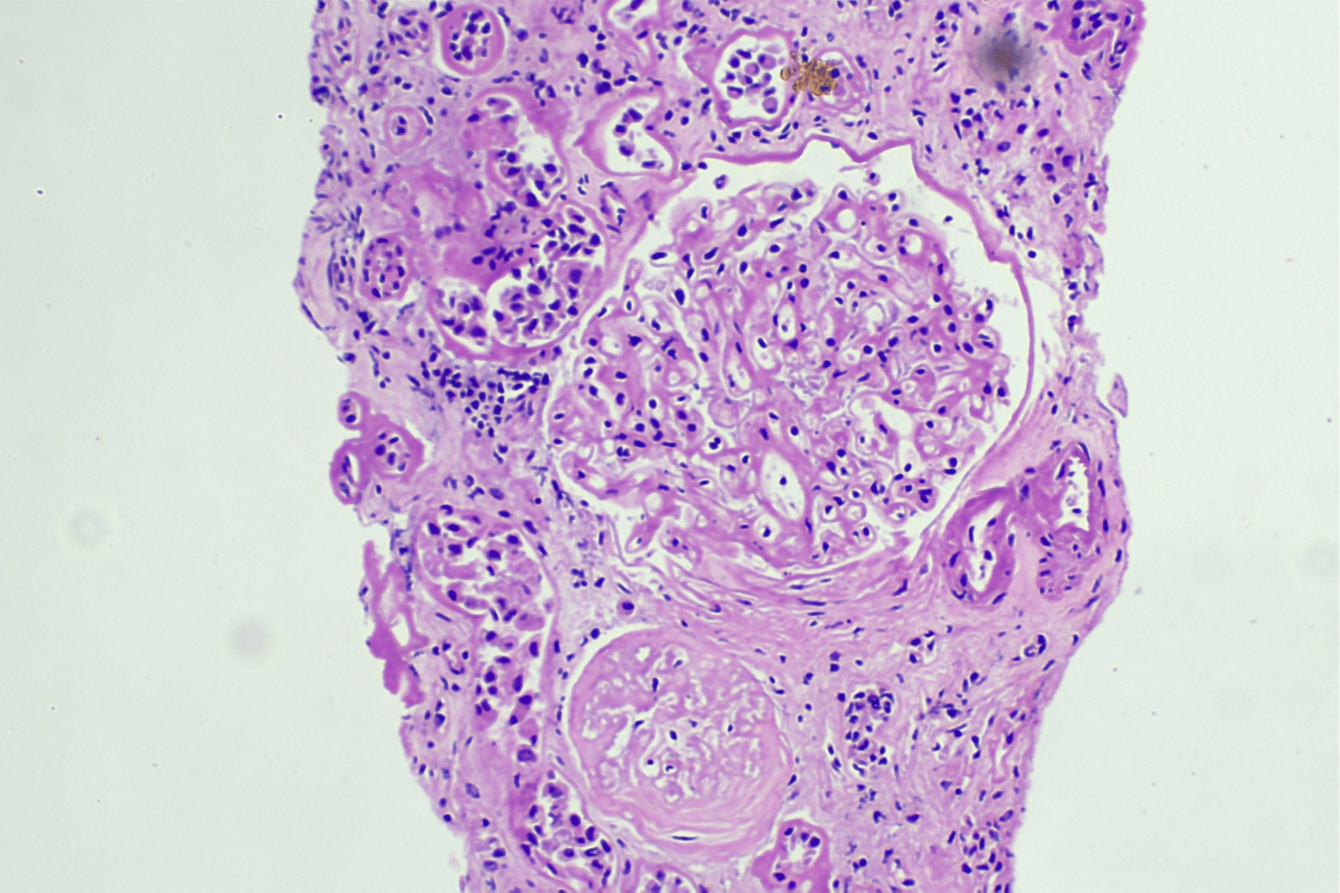
Renal cortex with interstitial infiltrates Low-power photomicrograph showing renal cortex with dense interstitial lymphoplasmacytic infiltrates, moderate tubular atrophy, and scattered tubules exhibiting regenerative changes. Multiple RBC and WBC casts are visible within tubular lumens. No glomerular crescents or endocapillary hypercellularity are noted (H&E, ×200). WBC: white blood cell; RBC: red blood cell; H&E: hematoxylin and eosin stain

**Figure 2:**
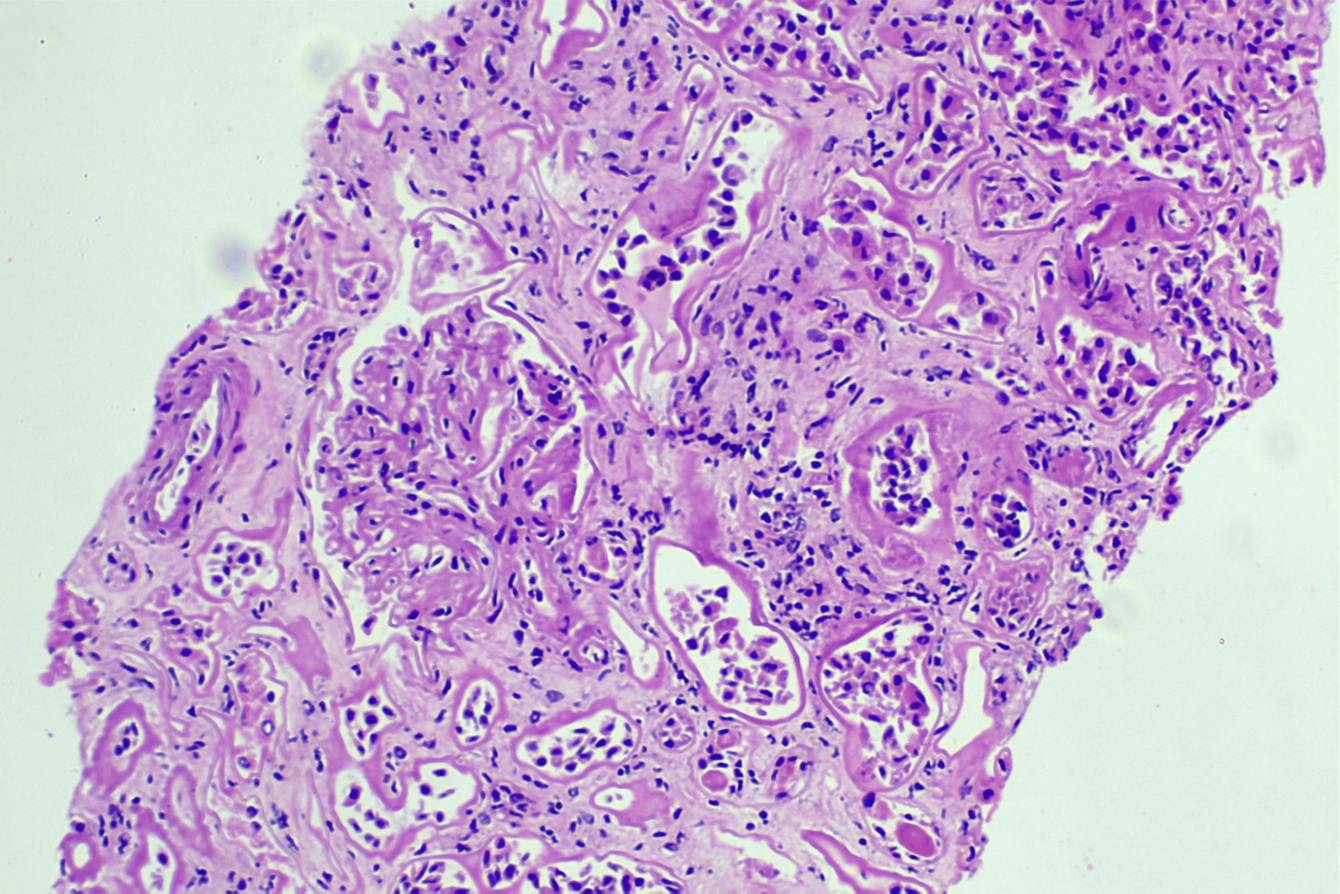
Glomerulus with mesangial widening High-power photomicrograph showing a glomerulus with mild mesangial widening, adjacent to interstitium exhibiting chronic inflammatory infiltrates and tubulointerstitial injury. No segmental sclerosis, necrosis, or immune complex deposition is visible. Features are consistent with acute on chronic interstitial nephritis (H&E, ×400). H&E: hematoxylin and eosin stain

Because the patient had a rapidly rising creatinine, active sediment, and sub-nephrotic proteinuria, findings atypical for diabetic kidney disease and concerning for RPGN, a renal biopsy was performed. Light microscopy revealed four glomeruli, two of which were globally sclerosed. The remaining glomeruli showed only mild mesangial widening without crescents, necrosis, or endocapillary proliferation. The interstitium demonstrated dense lymphoplasmacytic infiltrates, moderate tubular atrophy, regenerative atypia, and the presence of RBC and WBC casts. Approximately 15%-20% interstitial fibrosis was noted. Direct immunofluorescence was negative for immunoglobulins and complement. Representative biopsy findings are shown in Figure 1 and Figure 2. These findings confirmed a diagnosis of acute on chronic interstitial nephritis rather than glomerulonephritis.

## Discussion

AIN is a well-recognized but often underdiagnosed cause of acute kidney injury. Its clinical features are highly variable and may overlap with glomerular diseases when patients present with proteinuria, hematuria, and urinary casts [1,2]. In such settings, the clinical picture may closely resemble RPGN, a condition that requires urgent immunosuppressive therapy [3,4]. This overlap creates a diagnostic challenge with major therapeutic implications.

In patients with diabetes mellitus, diagnostic complexity increases further. Kidney dysfunction is frequently attributed to diabetic nephropathy, and when renal function deteriorates acutely with nephritic sediment, RPGN is often suspected [5]. However, such assumptions can delay correct diagnosis and risk unnecessary exposure to aggressive therapy. In our patient, the combination of rapidly rising creatinine, nephritic urinary findings, and oliguria initially suggested RPGN, but renal biopsy revealed acute on chronic interstitial nephritis instead. This distinction was critical, as treatment strategies for AIN differ markedly from those for glomerulonephritis [2,6].

This case highlights the indispensable role of renal biopsy in evaluating patients with atypical or rapidly progressive renal impairment. Reliance on urinalysis and clinical suspicion alone may lead to misclassification and inappropriate therapy. Histological confirmation not only avoids unnecessary immunosuppression in non-glomerular disease but also facilitates timely recognition and management of reversible conditions such as AIN [7,8].

By underscoring this diagnostic pitfall, the report emphasizes the importance of maintaining a broad differential in diabetic patients presenting with acute kidney injury. Clinicians should remain alert to interstitial causes, particularly when the clinical course deviates from the classical pattern of diabetic nephropathy.

## Conclusions

AIN can closely mimic RPGN, particularly in diabetic patients who present with acute kidney injury and nephritic urinary sediment. Attributing such presentations solely to diabetic nephropathy or glomerulonephritis may lead to misdiagnosis and inappropriate management.

This case emphasizes the indispensable role of kidney biopsy in distinguishing between glomerular and interstitial pathology. Early histological confirmation prevents unnecessary immunosuppression in non-glomerular disease and ensures that reversible conditions such as interstitial nephritis are promptly identified. Clinicians should remain vigilant for atypical presentations in diabetic patients, maintaining a broad differential diagnosis and considering renal biopsy when the clinical course does not fit the expected pattern.
